# Ensemble classification of autism spectrum disorder using structural magnetic resonance imaging features

**DOI:** 10.1002/jcv2.12042

**Published:** 2021-11-06

**Authors:** Yanli Zhang‐James, Jan K. Buitelaar, The ENIGMA‐ASD Working Group, Daan van Rooij, Stephen V. Faraone

**Affiliations:** ^1^ Department of Psychiatry and Behavioral Sciences SUNY Upstate Medical University Syracuse New York USA; ^2^ Radboudumc, Radboud University Medical Center Nijmegen The Netherlands; ^3^ Donders Institute for Brain, Cognition, and Behaviour Radboud University Medical Center Nijmegen The Netherlands; ^4^ Department of Cognitive Neuroscience Radboud University Medical Center Nijmegen The Netherlands; ^5^ Donders Centre for Cognitive Neuroimaging Radboud University Medical Center Nijmegen The Netherlands; ^6^ Department of Neuroscience and Physiology SUNY Upstate Medical University Syracuse New York USA

**Keywords:** autism spectrum disorder, biomarkers, classification, machine learning, MRI

## Abstract

**Background:**

Autism spectrum disorder (ASD) is characterized by a spectrum of social and communication impairments and rigid and stereotyped behaviors that have a neurodevelopmental origin. Although many imaging studies have reported structural and functional alterations in multiple brain regions, clinically useful diagnostic imaging biomarkers for ASD remain unavailable.

**Methods:**

In this study, we applied machine learning (ML) models to regional volumetric and cortical thickness data from the largest structural magnetic resonance imaging (sMRI) dataset available from the Enhancing Neuro Imaging Genetics Through Meta‐Analysis (ENIGMA) consortium (1833 subjects with ASD and 1838 without ASD; age range: 1.5–64; average age: 15.6; male/female ratio: 4.2:1).

**Results:**

The highest classification accuracy on a hold‐out test set was achieved using a stacked Extra Tree Classifier. The area under the receiver operating characteristic (ROC) curve (AUC) was 0.62 (95% confidence interval [CI]: 0.57, 0.68) and the area under the precision‐recall curve was 0.58. Learning curve analysis showed the good fit of the model and suggests that more training examples will not likely benefit model performance.

**Conclusions:**

Our results suggest that sMRI volumetric and cortical thickness data alone may not provide clinically sufficient useful diagnostic biomarkers for ASD. Developing clinically useful imaging classifiers for ASD will benefit from combining other data modalities or feature types, such as functional MRI data and raw images that can leverage other machine learning (ML) techniques such as convolutional neural networks.

## Graphical abstract

There are currently no clinically useful, reliable and reproducible MRI classifiers for ASD. Prior studies with small sample sizes often reported inflated estimates from overfitted models. Our ensemble classifier using the largest structural MRI dataset from the ENIGMA consortium showed good fit and achieved a modest but significant classification AUC of 0.62 on a hold‐out test set.

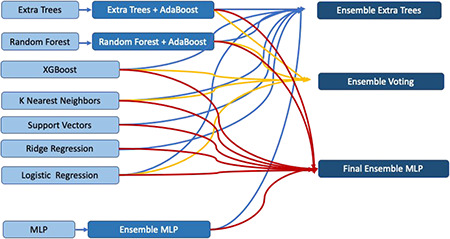

**Key points**

- There are currently no clinically useful, reliable, and reproducible diagnostic magnetic resonance imaging (MRI) classifiers for autism spectrum disorder
- Prior studies with small sample sizes often reported inflated estimates from overfitted models
- Our ensemble classifier using the largest structural MRI dataset from the Enhancing Neuro Imaging Genetics Through Meta‐Analysis consortium showed good fit and achieved a significant but modest classification AUC of 0.62 on a true hold‐out test set
- Improving classifier's performance will need additional features, for example, by combining different MRI data modalities

## INTRODUCTION

Autism spectrum disorder (ASD) is a common neurodevelopmental condition affecting roughly 1 in 160 of children worldwide (Elsabbagh et al., 2012) and 1 in 54 in the United States (Maenner et al., 2020; https://www.autism‐society.org). ASD is associated with socioeconomic burden and health care costs (Hong et al., 2020; Leibson et al., 2020; Lord et al., 2006; Schofield et al., 2019). Currently, ASD is diagnosed solely by trained experts with subjective clinical assessments of the behavior, including hallmarks in social interaction, communication, and repetitive behaviors (Emerson et al., 2017; Hazlett et al., 2017; Wolff et al., 2018). Developing objective diagnostic measures holds great potential for reliable and early identification of children with ASD, allowing for crucial interventions at earlier ages (Ratajczak, 2011). In addition, such objective and biological diagnostics may provide information underlying the pathophysiology and potential targets for therapeutic interventions, thus helping to guide the treatments and monitor the responses (Perez et al., 2014).

Many biomarkers have been evaluated, including genomics and transcriptomics, hormones, metabolites, brain magnetic resonance imaging (MRI), and others. None, however, were supported by sufficient evidence for clinical uses (Goldani et al., 2014; Ratajczak, 2011; Shen et al., 2019). There are currently no reliable and reproducible MRI biomarkers that can discriminate individuals with and without ASD with both high sensitivity and specificity (Pagnozzi et al., 2018). Nevertheless, structural and functional differences identified between the brains of individuals with ASD and those without (Boedhoe et al., 2020; Dichter, 2012; van Rooij et al., 2018) have continued to motivate the development of novel multivariate methods. Indeed, a neurodevelopmental origin with multifactorial dysfunctional networks has been supported by neuroimaging (Emerson et al., 2017; Hazlett et al., 2017; Wolff et al., 2018) and genomic studies of ASD (Grove et al., 2019). Among those novel multivariate methods, machine learning (ML) has gained much popularity in recent years.

ML diagnostic classifiers have proven to be highly accurate and clinically useful for some disorders such as diabetic eye disease (Beede et al., 2020). However, the clinical utilities of reported MRI‐based ML classifiers for ASD remain unclear. Most prior ML studies for ASD have used extremely small sample sizes (<100) (Eslami et al., 2021; Wolfers et al., 2015). Given the highly heterogeneous and complex nature of ASD, much larger samples are needed for stable and accurate estimates (Brain & Webb, 1999; Pulini et al., 2019; Vabalas et al., 2019; Wolfers et al., 2015, 2019). To facilitate sample sharing for ASDs, the Autism Brain Imaging Data Exchange (ABIDE) initiative collected and aggregated functional and structural brain imaging data from multiple sites located all over the world. Studies utilizing ABIDE datasets reached sample sizes over 1000. However, even sample sizes of this range are still “small” for most ML applications and small sample sizes are known to be negatively associated with reported accuracies, suggesting inflated estimates from overfitted models (Vabalas et al., 2019; Wolfers et al., 2015; Zhang‐James et al., Preprint). Furthermore, almost all ML studies of ASD used some type of cross‐validation to assess accuracy rather than an independent test set. Estimates of accuracy based on cross‐validation alone are known to yield overly optimistic estimates of accuracy (Brain & Webb, 1999; Wolfers et al., 2015).

Most of the studies using the ABIDE dataset focused on fMRI data; only a handful examined structural MRI (sMRI) features. Among those studies that used very small sample sizes (<300 training samples), they reported a wide range of accuracies from 68%–99% (reviewed by Eslami et al., 2021). A few had larger samples sizes ranging from 650 to 906 (Demirhan, 2018; Haar et al., 2016; Katuwal et al., 2015), all of which reported lower classification accuracies (<60%). With the most recent effort led by the Enhancing Neuro Imaging Genetics Through Meta‐Analysis (ENIGMA) ASD Working Group (WG), a larger collaborative dataset with over 3000 sMRI samples has been created. This dataset incorporated the sMRI data from the previous ABIDE datasets and aggregated additional contributions from around the world. To harmonize MRI data acquired from different sites and instruments, and to overcome the limitations of different local regulations on sharing the raw MRI image, the ENIGMA ASD‐WG adopted standardized processing protocols at each contributing site and generated the aggregated tabular data for dissemination. In prior work using standard statistical methods, this large sample size provided more power to detect small effects (van Rooij et al., 2018). In this paper, we use this large dataset to determine whether ML models can discriminate the brains of individuals with and without ASD.

## MATERIALS AND METHODS

### MRI samples

The current study was approved by all contributing members of the ENIGMA‐ASD Working Group. Each participating site had approval from its local ethics committee to perform the study and to share de‐identified, anonymized individual data. T1‐weighted structural MRI (sMRI) data from 3671 subjects from 56 acquisition sites (by October 2019) were processed using the consortium's standard segmentation algorithms in FreeSurfer (V5.1 and V5.3) (Hoogman et al., 2017). One hundred and fifty‐five geometrical features were used including 35 cortical surface area, 35 cortical thickness measurements, and 7 subcortical regions from each hemisphere, and intracranial volume (ICV). One hundred and three subjects were removed due to missingness in more than 50% of variables resulted from segmentation failure. One outlier observation, with its value outside of 1.5 times the interquartile range (iqr 1.5), was removed. Remaining subjects with missing observations (*N* = 770, 21.6% of the total sample) accounted for 1.87% of the total missing observations (ranging from 0.5% to 3.74% missingness for each variable). We imputed these missing observations using multiple imputation with chained equations with linear regression in STATA16. Twenty imputed datasets were inspected and missing values were replaced with the mean of the imputed values.

Using methods described previously (Zhang‐James et al., 2021), we randomly assigned data to training (∼70%), validation (∼15%), and test (∼15%) subsets that have equal representation of diagnosis, sex, age subgroup (child < 18 vs. adult), and acquisition sites. Eighty‐eight additional samples were excluded because they were from a site and subgroup that had only cases (67) or only controls (21). Table S1 shows the sample assignment by site.

Next, we balanced the training set for the case and control groups within each sex, age, and site subgroup by random oversampling of the under‐represented diagnostic group, a procedure commonly used to deal with class imbalance (Menardi & Torelli, 2014). The resulting balanced training set is described in Table 1. The validation and test sets were not balanced by age, sex, and site; however, due to our sample splitting procedures, they contain the same demographic samples as the training set. This was done to ensure that the learned classification was not biased by difference of sample demographic composition and of acquisition site and could be generalized to validation and test sets.

**TABLE 1:** Balanced training set

Diagnosis	*F*	*M*	Total
Control			
Mean age	13.6	15.8	15.3
Std of age	7.2	8.7	8.4
*N*	320	1224	1544
ASD			
Mean age	13.7	15.6	15.2
Std of age	7.6	8.4	8.3
*N*	320	1224	1544
Total			
Mean age	13.6	15.7	15.2
Std of age	7.4	8.5	8.3
*N*	640	2448	3088

### Feature preprocessing

Because the 155 sMRI geometrical features are highly correlated with one another, we investigated two methods for data‐dimensionality reduction. Using principal component analysis (PCA) on the training set features, we obtained 35 components that explained 80% of total variance. We then obtained these 35 component scores for the training, validation, and test samples and used them as features in all the ML classifiers.

As an alternative method to obtain a reduced set of features, we trained an autoencoder (AE) consisting of two layers of fully connected neural networks in both the encoder and decoder layers. The AE latent feature space encodes the compressed information contained in the original features. A multiple layer AE maps the correlated features into components that are non‐linearly related to the initial features. Our AE was implemented using Keras API (version 2.3.1) and the TensorFlow library (version 1.14.0). We implemented HyperOpt (Bergstra et al., 2013) to tune the numbers of units in each layer (4–500) and a dropout rate for each layer (0.1–0.9). We also tested different gradient descent optimization algorithms with various learning rates (0.00001–0.01) and different batch sizes (4–256). The AE models were trained and tuned using mean squared error (MSE) between the original features and the re‐constructed features as the loss function. The best model was chosen based on the lowest MSE for the validation set. The best fitting model compressed the original 155 features into 48 latent features.

### Ensemble classifiers for ASD

Eight different base classifiers were investigated including random forest classifier (RF), extra trees classifier (ETC), k‐nearest neighbors (KNN), support vector machine with a linear kernel (LSV), Ridge regression (Ridge), logistic regression (Logistic), XGBoost (XGB), and a multilayer perceptron classifier (MLP). MLP was implemented in Keras (version 2.4.0) and TensorFlow V2.4.1. XGB was implemented using Scikit‐Learn wrapper intervace for XGB. All other classifiers were implemented using Scikit‐Learn library V0.24.

**FIGURE 1:**
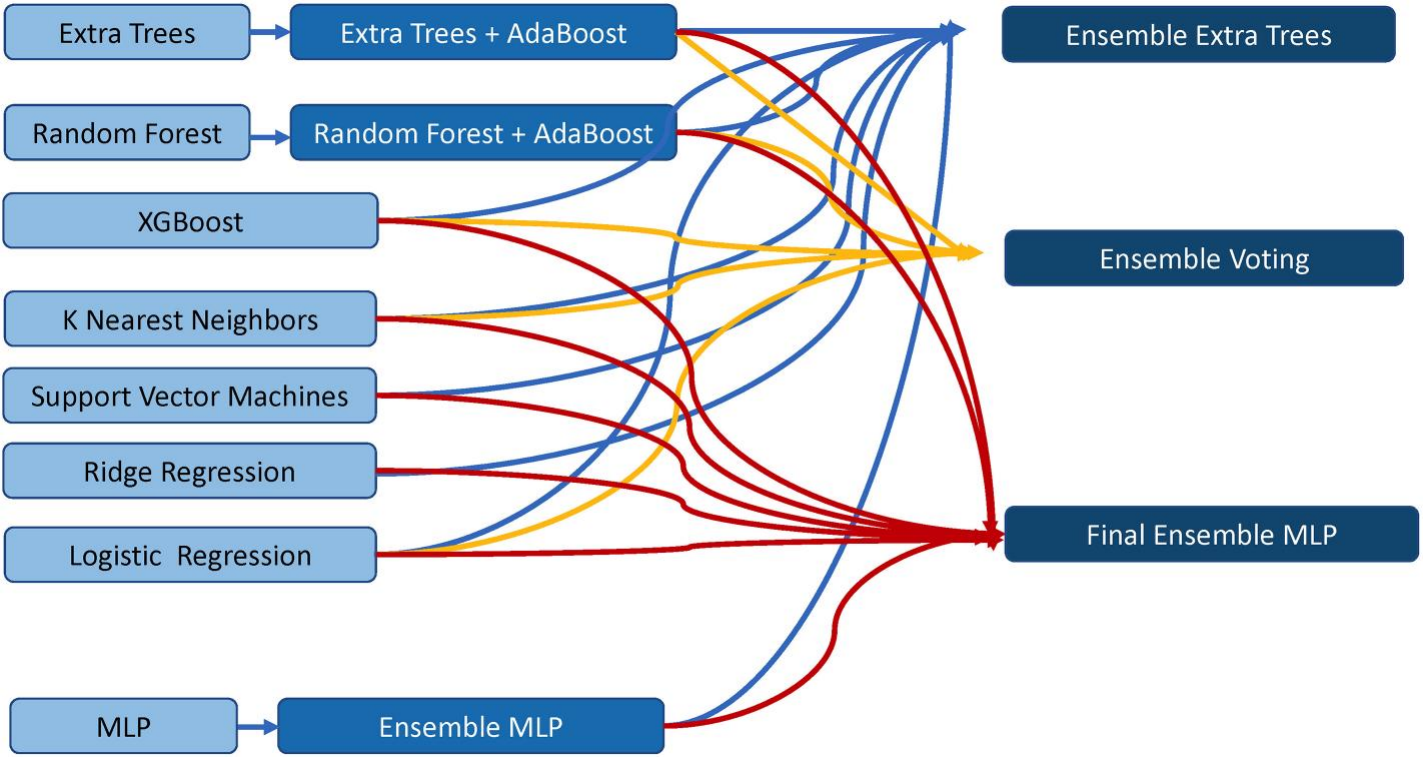
The ensemble machine learning pipeline includes eight base models that were trained as independent classifiers (light blue), followed by various ensemble methods in the second tier (dark blue) to improve the individual models, and the cross‐classifier ensemble in the third tier (navy blue) to combine multiple different classifiers

Ensemble or boosting methods were applied to some of the base models to improve their performance. This includes ensemble MLP as we described previously (Zhang‐James et al., 2021), and the Adaboost algorithm applied to RF and ETC. A final ensemble approach was constructed across multiple different classifiers. We combined five different base models (RF, ETC, XGB, KNN, and Logistic) into an ensemble voting classifier. We also used the output probabilities from all eight classifiers (or their ensemble or boosted models) as new features, and trained another extra tree classifier as a final stacked Ensemble‐ETC, and a final stacked Ensemble‐MLP. The overall hierarchical ML ensemble classifier pipeline is depicted in Figure 1.

The receiver operating characteristic (ROC) curve plots sensitivity over the full range of false positive rates (equivalent to 1‐specificity). The area under the ROC (AUC) measures the overall diagnostic accuracy of a classifier. AUC is a preferred metric for imbalanced dataset given that it is not biased toward models that perform well on the majority class at the expense of the minority class (He & Ma, 2013). The standard error and asymptotic normal confidence intervals (CI) of the AUC were computed using DeLong's algorithm (DeLong et al., 1988). In addition to the AUC, we also report the area under the precision‐recall curve (AUPRC) for the final model. The precision‐recall curve plots precision (the percentage of examples classified as positive that are true positive, also known as positive predictive value, PPV) over recall (sensitivity). The AUPRC is superior and more informative than the AUC for assessing extremely imbalanced datasets (Davis & Goadrich, 2006).

We fine‐tuned the MLP‐based classifier using HyperOpt. Hyperparameter tuning for all other classifiers used Scikit‐Learn's grid search algorithm (Pedregosa et al., 2012). We used the area under the ROC curves as a measure of accuracy. To avoid overfitting, we always chose the model with the highest validation AUC but lowest training AUC. Classifiers that did not yield statistically significant validation AUCs (with 95%CI not above 0.5, a random non‐discriminative level), such as decision tree and Nu‐Support Vector Machines, are not included in this report. All classifiers were tuned on the training and validation sets. Only the best hyperparameter sets were tested on the hold‐out test samples for all the base models and ensemble models.

Finally, we use learning curve analysis to evaluate the model's bias and variance, as well as the sample size effect to draw inferences about how models might be improved in the future (Webb et al., 2011). The learning curve analysis was carried out by using the Scikit‐learn function *learning_curve*, in which deciles of the total sample were used to fit the model. For each decile of sample size, we plotted the training and the 10‐fold cross‐validation AUCs.

### Feature importance scores

Feature Importance scores (FI) were computed first for the 35 PCA components from each of the base models. The FI scores for the original 155 geometrical features were subsequently obtained by summing the products of their absolute values of the loading in each component and the corresponding FI scores of the component. For RF, ETC, and XGB, FI was computed as the mean decrease of impurity within each tree and provided directly by the fitted attribute “*feature_importances_”* in Scikit‐Learn. For KNN, FI was obtained by computing the difference between the baseline metric and metric from each permutated feature column via the fitted estimator “permutation_importance” in Scikit‐Learn. For LSV, Ridge, and Logistic regression classifiers, coefficients were used. For MLP, a game theory‐based method, SHapley Additive exPlanations (SHAP) values were computed (Lundberg & Lee, 2017).

The FI scores were rescaled to facilitate the comparisons and correlation evaluation across the different models by dividing each score over the sum of all 155 FI scores.

All ML algorithms were written in Python 3.6. The ML codes used in this study are readily available to the public via the GitHub repository (https://github.com/ylzhang29/ML_DL_Framework).

## RESULTS

The final dataset consisted of 50% non‐ASD controls (*n* = 1788%, 77% male) and 50% ASD participants (*n* = 1780%, 85% male). Ages ranged from 1.5 to 63 years old, with 71% children (age < 18 years) and 29% adults (age ≥ 18 years). In the unbalanced total dataset, ASD diagnosis was significantly biased by sex (*X* ^2^_(1)_ = 46.9, *p* < .0001) and sites (*X* ^2^_(19)_ = 87.5, *p* < .0001), but not by the overall age. In the balanced training set, ASD diagnosis was no longer associated with either sex, site, or age.

**FIGURE 2:**
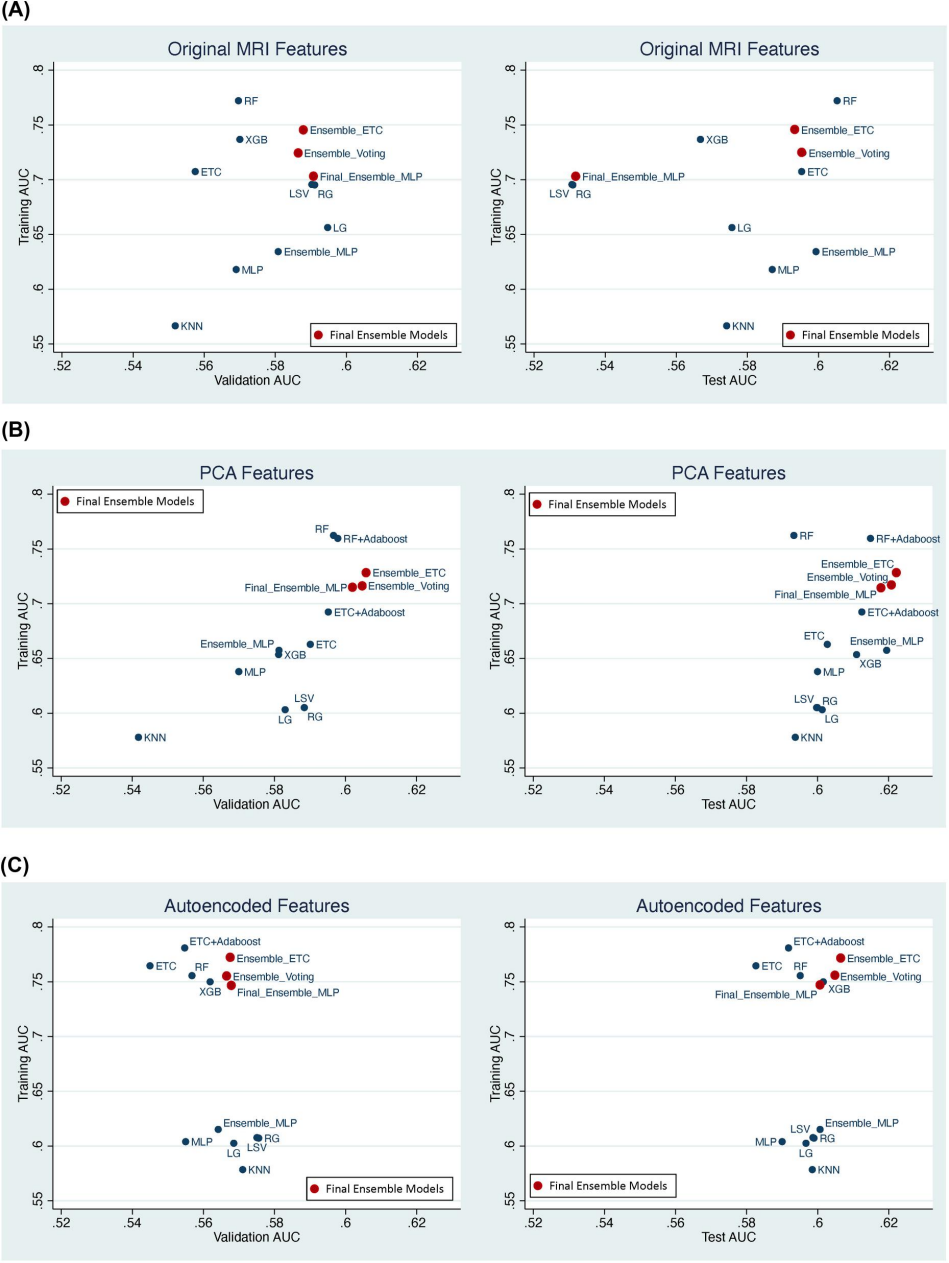
Final training, validation and test AUCs were plotted for each classifier using different features. (A) magnetic resonance imaging features. (B) Principal component analysis features. (C) Autoencoded features

Figure 2A shows results based on the original 155 sMRI features. The left panel plots the training AUC versus the validation AUC from the best model of each base classifier and the ensemble classifiers. These models were then tested on the hold‐out test set and the test AUCs were plotted against the corresponding training AUCs in the right panel. Figure 2B,C shows the corresponding results for PCA features and autoencoded features

Overall, the training AUCs were not different among the models using different types of features. However, models using PCA features resulted in the highest validation and test AUCs in comparison with those using the original sMRI features or autoencoded features (Figure S1). The validation AUCs in models using the PCA features were significantly higher than those that used autoencoded features (Wilcoxon signed‐rank test *z* = 2.7, *p* = .005), although not statistically different from those using original MRI features. For test AUCs, the use of PCA features significantly improved AUCs compared with either the MRI features (Wilcoxon signed‐rank test *z* = 2.9, *p* = .004), or the autoencoded features (*z* = 2.6, *p* = .006).

The highest test AUC was obtained by the Ensemble ETC using the PCA features (AUC 0.624, 95%CI: 0.57, 0.68). The ROC curve is shown in Figure S2A. The AUPRC was 0.58 (Figure S2A,B). The learning curve analysis of the model is shown in Figure S2C with the training and 10‐fold cross validation AUCs converging effectively when training examples increase.

We examined the classification AUCs across different sites. Figure S3 shows the forest plots of the training and test set AUCs and their 95%CIs for each site. The heterogeneity was highly significant across sites measured by the I2 statistics which was 70.1% (*p* < .001) for the training AUCs and 67.1% for the test AUCs. We also found that the confidence interval ranges of both AUCs were negatively associated with training sample size of their sites, fitting an exponential decay curve.

The test AUCs were also significantly different between children and adults 0.66 (95%CI: 0.60–0.72) in children versus 0.52 (95%CI: 0.41–0.63) in adults, *X* ^2^_(1)_ = 5.19, *p* = .02). Test AUCs for male and females were 0.63 (95%CI: 0.57–0.68) versus 0.57 (95%CI: 0.43–0.70), but not statistically different (*X* ^2^_(1)_ = 0.69, *p* = .4).

Feature importance scores are listed in the Table S2. We found that scores from different base models were all highly and statistically significantly correlated (Pearson's *r* range from 0.42 to 0.99, *p* < .0001). The highest correlations were among the group of non‐linear models and those of the linear models (see the correlation heatmap in Figure S4A). A scatter plot of the mean scores of non‐linear versus the linear models for the individual brain MRI features was shown in the Figure S4B, highlighting the overall highly significant correlation (*r* = 0.51, *p* < .0001) and the most important MRI features used in each models.

## DISCUSSION

We developed a multi‐tiered ensemble classification pipeline for regional volumetric and cortical thickness sMRI data in the service of deriving a classification scheme that would successfully discriminate structural brain features from individuals with ASD versus matched controls. Despite using the largest sMRI dataset available from the ENIGMA ASD working group, classification accuracies were modest, but significantly above chance. The highest classification AUC, obtained by using a stacked ensemble extra tree model was 0.62 (95%CI: 0.57, 0.68). In addition, we showed that PCA transformation of the processed sMRI features was useful in reducing the numbers of highly correlated features and improving classification accuracies.

Our study improves upon prior ML studies of ASD in several ways. First, because of the large sample size, we were able to construct the training, validation, and hold‐out test sets for model development and evaluation. Prior studies using smaller samples did not use independent tests sets (Eslami et al., 2021; Wolfers et al., 2019), which may have led to overly optimistic estimates of accuracy (Brain & Webb, 1999; Wolfers et al., 2015). Indeed, studies with very small sample sizes (<300 training samples) often reported higher accuracies with a wide range of variability (e.g., 68%–99% as reviewed by Eslami et al. (2021) than those that used larger samples sizes (e.g., sample sizes ranging from 650 to 906 in Demirhan (2018), Haar et al. (2016), and Katuwal et al. (2015). None of the above studies with larger sample sizes reported classification accuracies higher than 60% even with using various forms of cross‐validation. In our study, model hyperparameter tuning used the training and validation sets with a completely independent test set to compute final estimates of accuracy. This approach prevents the data leakage and overfitting (Zhang‐James et al., 2021; Zhang‐James et al., Preprint). Therefore, our modest but significant test AUC represent an improved and more reliable estimate of the classification accuracy for ASD.

Second, despite the data aggregation from many contributing sites and data heterogeneity, our sample splitting process guaranteed that the training, validation, and testing sets had the same demographic and site compositions. Unlike the leave‐one‐site‐out strategy, our sampling strategy ensured that any feature representation learned from the training set could reliably be generalized to the validation and test sets. Third, during the hyperparameter search, we chose the models with high validation AUCs and low training AUCs. These choices are reflected in Figure 2A–C and suggest that there is no overfitting. Indeed, our learning curve analysis showed that the training and validation curves converged nicely, which is the pattern one expects when models do not overfit the data (Webb et al., 2011).

The total number of samples contributed from each site and demographic (sex and age) information influenced the classifier's performance across these subgroups. In addition to the site performance heterogeneity, we also observed different classification accuracies between children and adults. Because there were substantially more samples from children and male participants, those subgroups demonstrated significant classification AUCs, whereas the adult and female test AUCs were not statistically significant. It is also possible that there are larger anatomical differences between the ASD and control subjects for children and male groups. However, it is difficult to isolate the causes with the current sample size differences.

Interpretability of the ML models is a critical step towards to clinical utility in precision medicine. Our use of a multi‐tiered ensemble approach and feature transformation and reduction methods makes it difficult to clarify the overall importance of brain features in the final model. Furthermore, the relatively low overall prediction accuracies cautioned us from attempting any conclusive interpretation. Nevertheless, our feature importance analyses of all base models revealed interesting, highly correlated patterns despite of the drastic differences among the model algorithms and the feature importance methods. This result suggests that various widely different ML models, when optimized to their best, learn and utilize a highly similar set of the informative features for making predictions, highlighting the inherent patterns of differences between the ASD and control samples existing in the data. Indeed, among the most important features for both the linear and non‐linear models (labeled in red in Figure S4B), many were identified from previous univariate studies, such as subcortical volumes of putamen and accumbens, cortical thickness of the frontal and temporal regions (van Rooij et al., 2018). However, we also found some surface area measurements of highly important feature scores, particularly cingulate cortex and the banks of the superior temporal sulcus for the linear models. Prior studies have not found significant differences in cortical surface areas between ASD and the control subjects (van Rooij et al., 2018).

Our work has several limitations. First, because we combined data across many sites, we inherit all the limitations of the original studies. Heterogeneity of the data across contributing sites may reduce the sensitivity of the classifier due to added noise. This was evident in our cross‐site comparisons where highly significant heterogeneity of classification accuracies were found across different sites. Sites that contributed more samples were able to achieve more stable classification results than those contributed fewer samples. Nevertheless, considering that our learning curve analysis showed that approximately a minimum of 2500 training examples were needed for the model to converge, it suggests that individual samples from even sites with small sample sizes contributed to the model's overall learning of discriminating features. Heterogeneity of the data, however, may also provide advantage when developing a classifier for a highly heterogenous construct such as ASD.

Second, we only used volumetric and cortical thickness data. Adding additional informative features will likely help. One idea is to use other imaging modalities (such as functional MRI or Diffusion tensor imaging data). Another is to use three dimensional sMRI images to leverage the power of convolutional neural networks. However, as we know from the successful examples from computer vision, such applications will require a substantially large amount of data. Research communities, such as the ENIGMA consortium, will need to overcome limitations of the raw image sharing and aggregation.

Despite these limitations, we were able to detect case–control sMRI differences in individuals with ASD with a modest but significant AUC. The model interpretation highlighted some consistent findings from the previous studies, lending support for ML diagnostic classifiers for future clinically useful and interpretable diagnostic classifiers. However, our study also identified some roadblocks that we will need to overcome to achieve this goal, mainly the integration of multimodality of the MRI data and more sample sharing and aggregation to improve the sample size limitations, particularly those of females and adults which are currently underrepresented.

## CONFLICT OF INTERESTS

S.V.F. received income, potential income, travel expenses continuing education support, and/or research support from Takeda, OnDosis, Tris, Otsuka, Arbor, Ironshore, Rhodes, Akili Interactive Labs, Enzymotec, Sunovion, Supernus, and Genomind. With his institution, he has US patent US20130217707 A1 for the use of sodium‐hydrogen exchange inhibitors in the treatment of ADHD. He also receives royalties from books published by Guilford Press: Straight Talk about Your Child's Mental Health, Oxford University Press: Schizophrenia: The Facts and Elsevier: ADHD: Non‐Pharmacologic Interventions. He is Program Director of www.ADHDinAdults.com. He is a member of the Editorial Advisory Board for JCPP *Advances*. Y.Z‐J. is also a member of the Editorial Advisory Board for JCPP *Advances*. J.K.B. has been in the past 3 years a consultant to/member of advisory board of/and/or speaker for Takeda/Shire, Roche, Medice, Angelini, Janssen, and Servier. He is not an employee of any of these companies, and not a stock shareholder of any of these companies. He has no other financial or material support, including expert testimony, patents, and royalties. The remaining authors have declared that they have no competing or potential conflicts of interest to declare. [Corrections made on 22 June 2022, after first online publication: This Conflict of Interests statement has been updated in this version.]

## ETHICAL STATEMENT

The current study was approved by all contributing members of the ENIGMA‐ASD Working Group. Each participating site had approval from its local ethics committee to perform the study and to share de‐identified, anonymized individual data.

## AUTHOR CONTRIBUTIONS

Dr. Zhang‐James designed the experiments, carried out the modeling and analysis, and wrote the manuscript. Drs van Rooij, Buitelaar and Faraone also participated the experimental design, data analysis and writing of the manuscript. The ENIGMA ASD Working Group authors (see list) provided the site‐specific data and participated manuscript writing.

## Supporting information

Supporting Information S1

Table S2

## Data Availability

Data are publicly available through the ENIGMA ASD working group.

## APPENDIX 1

The ENIGMA‐ASD Working Group Author List:

Alessandra Retico, Beatriz Luna, Bob Oranje, Celso Arango, Christine Deruelle, Christine Ecker, Christine M. Freitag, Declan G.M. Murphy, Devon Shook, Eileen Daly, Fabio L.S. Duran, Fengfeng Zhou, Filippo Muratori, Geraldo F. Busatto, Gregory L. Wallace, Guillaume Auzias, Ilaria Gori, Jackie Fitzgerald, Jane McGrath, Jennifer Fedor, Joost Janssen, Joseph A. King, Katya Rubia, Kirsten O'Hearn, Liesbeth Hoekstra, Louise Gallagher, Luisa Lázaro, Mara Parellada, Margot Taylor, Maria Jalbrzikowski, Marlene Behrmann, Meiyu Duan, Michela Tosetti, Pedro G.P. Rosa, Rosa Calvo, Sara Calderoni, Shlomi Haar, Stenfan Ehrlich

**Table:**

Name	Affiliation
Evdokia Anagnostou	Bloorview Research Institute, University of Toronto, Toronto, Canada
Celso Arango	Child and Adolescent Psychiatry Department, Gregorio Marañón General University Hospital, School of Medicine, Universidad Complutense, IiSGM, CIBERSAM, Madrid, Spain
Guillaume Auzias	Institut de Neurosciences de la Timone, UMR 7289, Aix Marseille Université, CNRS, Marseille, France
Marlene Behrmann	Department of Psychology, Carnegie Mellon University, Pittsburgh, Pennsylvania, USA
Sara Calderoni	IRCCS Stella Maris Foundation, Viale del Tirreno 331, 56128 Pisa, Italy
Rosa Calvo	Department of Child and Adolescent Psychiatry and Psychology Hospital Clinic, Barcelona CIBERSAM, Universitat de Barcelona
Eileen Daly	Department of Forensic and Neurodevelopmental Sciences, Institute of Psychiatry, Psychology & Neuroscience King's College London, London, UK
Christine Deruelle	Institut de Neurosciences de la Timone, UMR 7289, Aix Marseille Université, CNRS, Marseille, France
Adriana Di Martino	Institute for Pediatric Neuroscience, NYU Child Study Center, New York, USA
Ilan Dinstein	Department of Psychology, Ben‐Gurion University of the Negev, Beer Sheva, Israel
Sarah Durston	Brain Center Rudolf Magnus, Department of Psychiatry, University Medical Center Utrecht, The Netherlands
Christine Ecker	Department of Child and Adolescent Psychiatry, Psychosomatics and Psychotherapy, University Hospital, Goethe University Frankfurt am Main, Frankfurt, Germany
Stephan Ehrlich	Division of Psychological and Social Medicine and Developmental Neurosciences, Faculty of Medicine, TU Dresden, Germany
Damien Fair	Department of Behavioral Neuroscience, Oregon Health & Science University, Portland, Oregon, USA
Jennifer Fedor	Department of Psychiatry, University of Pittsburgh, Pittsburgh, Pennsylvania, USA
Jackie Fitzgerald	Department of Psychiatry, School of Medicine, Trinity College, Dublin, Ireland
Christine M. Freitag	Department of Child and Adolescent Psychiatry, Psychosomatics and Psychotherapy, University Hospital, Goethe University Frankfurt am Main, Frankfurt, Germany
Louise Gallagher	Department of Psychiatry, School of Medicine, Trinity College, Dublin, Ireland
Ilaria Gori	National Institute for Nuclear Physics, Pisa Division, Largo B. Pontecorvo 3, 56124 Pisa, Italy
Shlomi Haar	Department of Brain and Cognitive Sciences, Zlotowski Center for Neuroscience, Ben‐Gurion University of the Negev, Beer Sheva, Israel
Liesbeth Hoekstra	Department of Cognitive Neuroscience, Donders Institute for Brain, Cognition and Behaviour, Donders Centre for Cognitive Neuroimaging, Radboud University Medical Centre, Nijmegen, The Netherlands
Maria Jalbrzikowski	Department of Psychiatry, School of Medicine, Trinity College, Dublin, Ireland
Joost Janssen	Child and Adolescent Psychiatry Department, Gregorio Marañón General University Hospital, School of Medicine, Universidad Complutense, IiSGM, CIBERSAM, Madrid, Spain
Joseph King	Division of Psychological and Social Medicine and Developmental Neurosciences, Faculty of Medicine, TU Dresden, Germany
Luisa Lázaro	Department of Child and Adolescent Psychiatry and Psychology Hospital Clinic, Barcelona CIBERSAM, Universitat de Barcelona, IDIBAPS
Jason Lerch	Mouse Imaging Centre, The Hospital for Sick Children, Toronto, Canada
Beatriz Luna	Department of Psychiatry, University of Pittsburgh, Pittsburgh, Pennsylvania, USA
Jane McGrath	Department of Psychiatry, School of medicine, Trinity College, Dublin, Ireland
Filippo Muratori	IRCCS Stella Maris Foundation, Viale del Tirreno 331, 56128 Pisa, Italy
Declan G.M. Murphy	The Sackler Institute for Translational Neurodevelopment, Institute of Psychiatry, Psychology & Neuroscience, King's College London, London, UK
Kirsten O’Hearn	Department of Physiology and Pharmacology, Wake Forest School of Medicine, Winston‐Salme, North Carolina, USA
Bob Oranje	Brain Center Rudolf Magnus, Department of Psychiatry, University Medical Center Utrecht, The Netherlands
Mara Parellada	Child and Adolescent Psychiatry Department, Gregorio Marañón General University Hospital, School of medicine, Universidad Complutense, IiSGM, CIBERSAM, Madrid, Spain
Alessandra Retico	National Institute for Nuclear Physics, Pisa Division, Largo B. Pontecorvo 3, 56124 Pisa, Italy
Pedro Rosa	Department of Psychiatry, Faculty of Medicine, University of São Paulo, São Paulo, Brazil. Center for Interdisciplinary Research on Applied Neurosciences (NAPNA), University of São Paulo, São Paulo, Brazil
Katya Rubia	Institute of Psychiatry, Psychology and Neuroscience, Kings College London, London, UK
Devon Shook	Brain Center Rudolf Magnus, Department of Psychiatry, University Medical Center Utrecht, The Netherlands
Margot Taylor	Diagnostic Imaging Research, The Hospital for Sick Children, University of Toronto, Canada
Michela Tosetti	IRCCS Stella Maris Foundation, Viale del Tirreno 331, 56128 Pisa, Italy
Gregory L. Wallace	Department of Speech and Hearing Sciences, The George Washington University, Washington, DC, USA
Fengfeng Zhou	College of Computer Science and Technology, and Key Laboratory of Symbolic Computation and Knowledge Engineering of Ministry of Education, Jilin University, Changchun, Jilin 130012, China
Sven Bolte	Center of Neurodevelopmental Disorders (KIND), Centre for Psychiatry Research; Department of Women's and Children's Health, Karolinska Institutet & Stockholm Health Care Services, Region Stockholm, Stockholm, Sweden
Janina Neufeld	Center of Neurodevelopmental Disorders (KIND), Centre for Psychiatry Research; Department of Women's and Children's Health, Karolinska Institutet & Stockholm Health Care Services, Region Stockholm, Stockholm, Sweden
Karl Lundin Remnélius	Center of Neurodevelopmental Disorders (KIND), Centre for Psychiatry Research; Department of Women's and Children's Health, Karolinska Institutet & Stockholm Health Care Services, Region Stockholm, Stockholm, Sweden
Kristiina Tammimies	Center of Neurodevelopmental Disorders (KIND), Centre for Psychiatry Research; Department of Women's and Children's Health, Karolinska Institutet & Stockholm Health Care Services, Region Stockholm, Stockholm, Sweden
Ciara Molloy	Department of Psychiatry, School of Medicine, Trinity College, Dublin, Ireland
Prof. Geraldo Busatto	Department of Psychiatry, Faculty of Medicine, University of São Paulo, São Paulo, Brazil. Center for Interdisciplinary Research on Applied Neurosciences (NAPNA), University of São Paulo, São Paulo, Brazil
Dr. Mauricio Martinho	Department of Psychiatry, Faculty of Medicine, University of São Paulo, São Paulo, Brazil. Center for Interdisciplinary Research on Applied Neurosciences (NAPNA), University of São Paulo, São Paulo, Brazil
Dr. Fabio Duran	Departamento de Neuropsiquiatria, UFSM—Universidade Federal de Santa Maria

### CONFLICT OF INTERESTS

Dr. Arango has been a consultant to or has received honoraria or grants from Acadia, Angelini, Gedeon Richter, Janssen Cilag, Lundbeck, Minerva, Otsuka, Roche, Sage, Servier, Shire, Schering Plough, Sumitomo Dainippon Pharma, Sunovion and Takeda. Dr. Anagnostou has served as a consultant or advisory board member for Roche and Takeda. Dr. Freitag has served as a consultant for Desitin regarding issues on ASD. Dr. Rubia has received funding from Takeda pharmaceuticals for another project. Dr. Gallagher received funding from the Meath Foundation and the National Children's Research Centre in Ireland. Dr. Parellada has served as a consultant, advisory board member or received honoraria from Sevier and Exeltis. She has received travel support from Janssen Cilag and Lundbeck. Dr. Murphy has served on advisory boards for Roche and Servier. Dr. Franke has received educational speaking fees from Medice. The other authors report no financial relationships with commercial interests.
