# Supplementary material for: Ensemble classification of autism spectrum disorder using structural magnetic resonance imaging features
Source: JCPP Adv. 2021 Nov 6;1(3):e12042. doi: 10.1002/jcv2.12042 (PMC10242907; doi:10.1002/jcv2.12042)
Supplement: Supplementary file 1 — Supporting Information S1 [file JCV2-1-e12042-s001.docx]

# Supporting Information

## Table S1. Training, validation and test samples from each site.

| Site | train | valid | test | excluded | Total | Source/Region/Country |
| --- | --- | --- | --- | --- | --- | --- |
| ABIDE | 648 | 175 | 111 | 7 | 941 | Mixed sources from US and EU |
| ABIDE2 | 664 | 179 | 122 | 59 | 1,024 | Mixed sources from US and EU |
| BRC | 35 | 10 | 7 | 0 | 52 | London, UK |
| Barcelo | 55 | 12 | 9 | 0 | 76 | Barcelona, SP |
| CMU | 17 | 6 | 4 | 0 | 27 | Pittsburg, USA |
| Dresden | 30 | 9 | 6 | 0 | 45 | Dresden, GE |
| FAIR | 56 | 16 | 12 | 0 | 84 | Portland, USA |
| FRANKFURT | 19 | 6 | 2 | 0 | 27 | Frankfurt, GE |
| FSM | 52 | 16 | 12 | 0 | 80 | Pisa, IT |
| MRC | 102 | 24 | 22 | 0 | 148 | London, UK |
| MYAD | 51 | 12 | 10 | 0 | 73 | Marseille, FR |
| PITT | 100 | 27 | 16 | 2 | 145 | Pittsburgh, USA |
| HGGM | 47 | 11 | 8 | 0 | 66 | Mardid, SP |
| SAOPAULO | 25 | 7 | 3 | 0 | 35 | Sao Paulo, BR |
| TCD | 60 | 19 | 10 | 0 | 89 | Dublin, IE |
| TORONTO | 257 | 60 | 51 | 8 | 376 | Toronto, CA |
| UMCU | 48 | 14 | 10 | 2 | 74 | Utrecht, NLD |
| Nijmegen 1 | 48 | 13 | 9 | 0 | 70 | Nijmegen, NL |
| Nijmegen 2 | 66 | 16 | 12 | 0 | 94 | Nijmegen, NL |
| Nijmegen 3 | 20 | 7 | 5 | 10 | 42 | Nijmegen, NL |
| Total | 2,400 | 639 | 441 | 88 | 3,568 |  |

Table S1. Sample assignments to training, validation and test sets.

## Fig S1. AUC comparison for different features.

FigS1. AUC comparison for different features. Box plots of training, validation and test AUCs for all base model and ensemble models are shown.

## Fig S2. Ensemble ETC model evaluation.


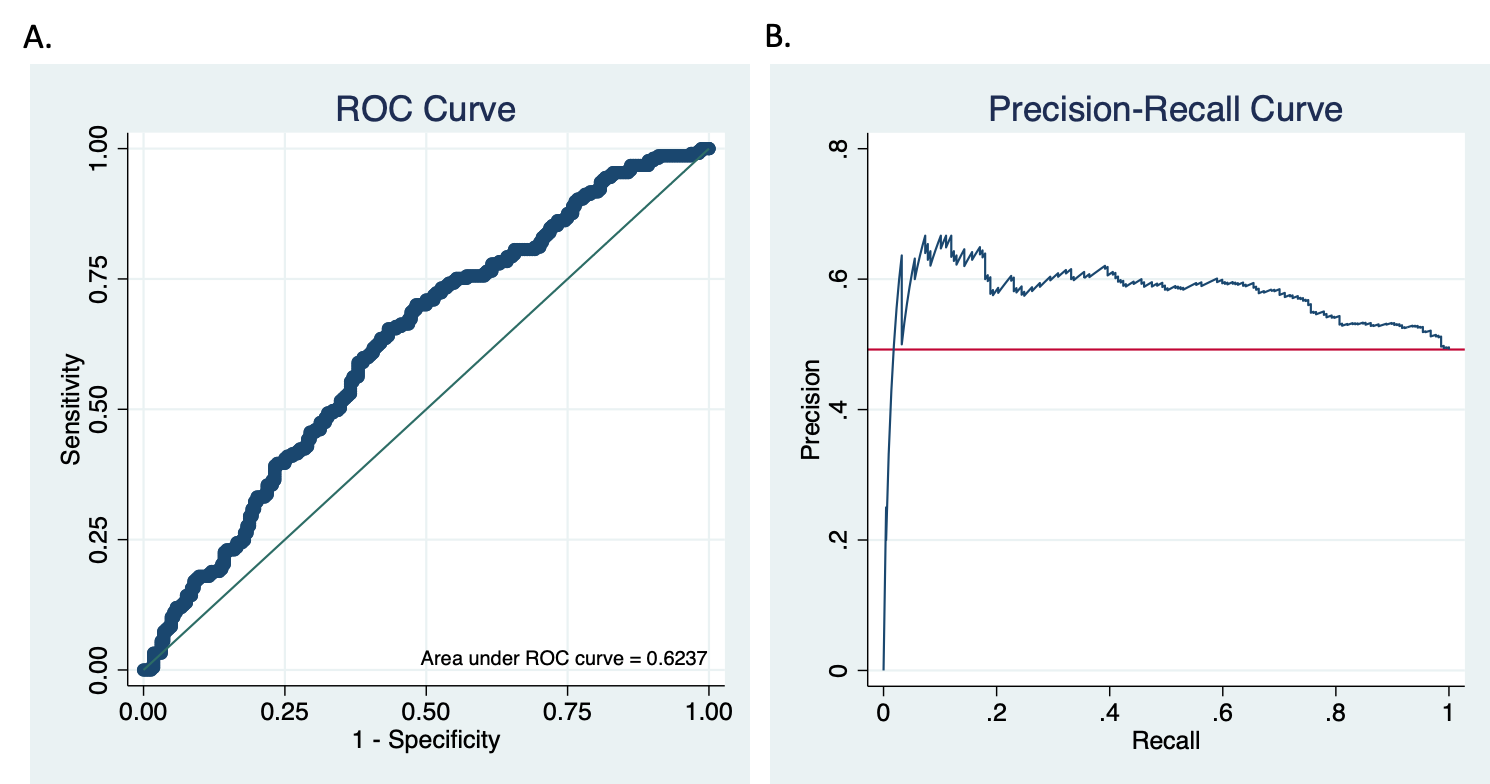


C.


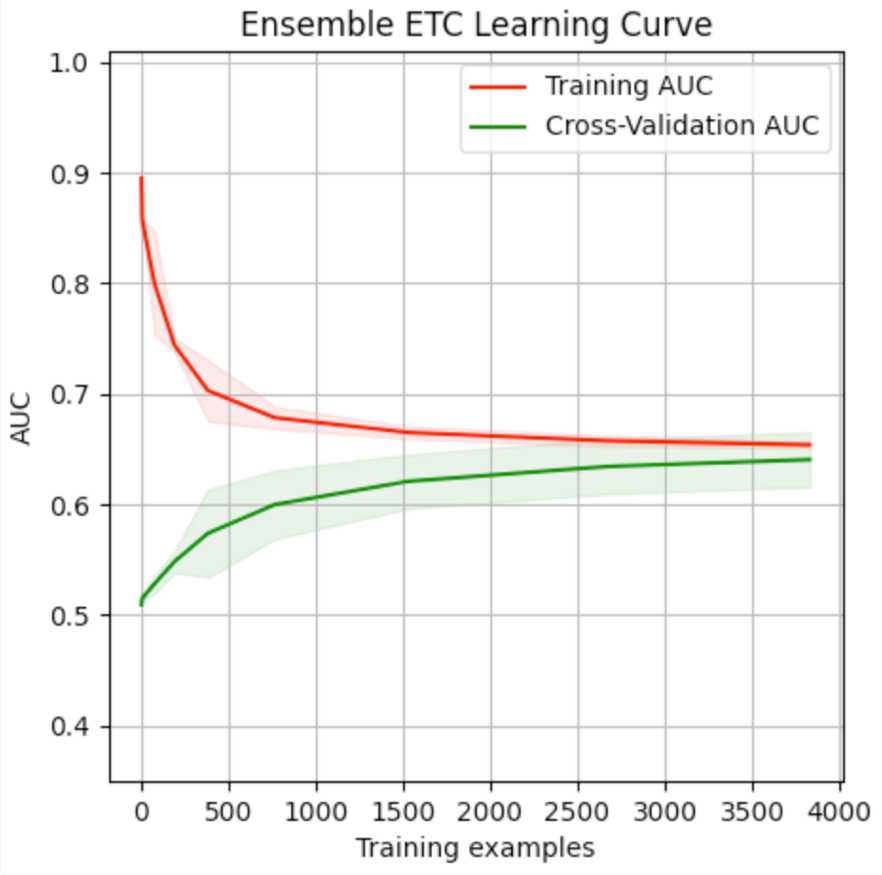


Fig S2. Stacked Final Ensemble ETC model evaluation. A. ROC curve. B. Precision-recall curve. C. Learning curve.

## FigS3.


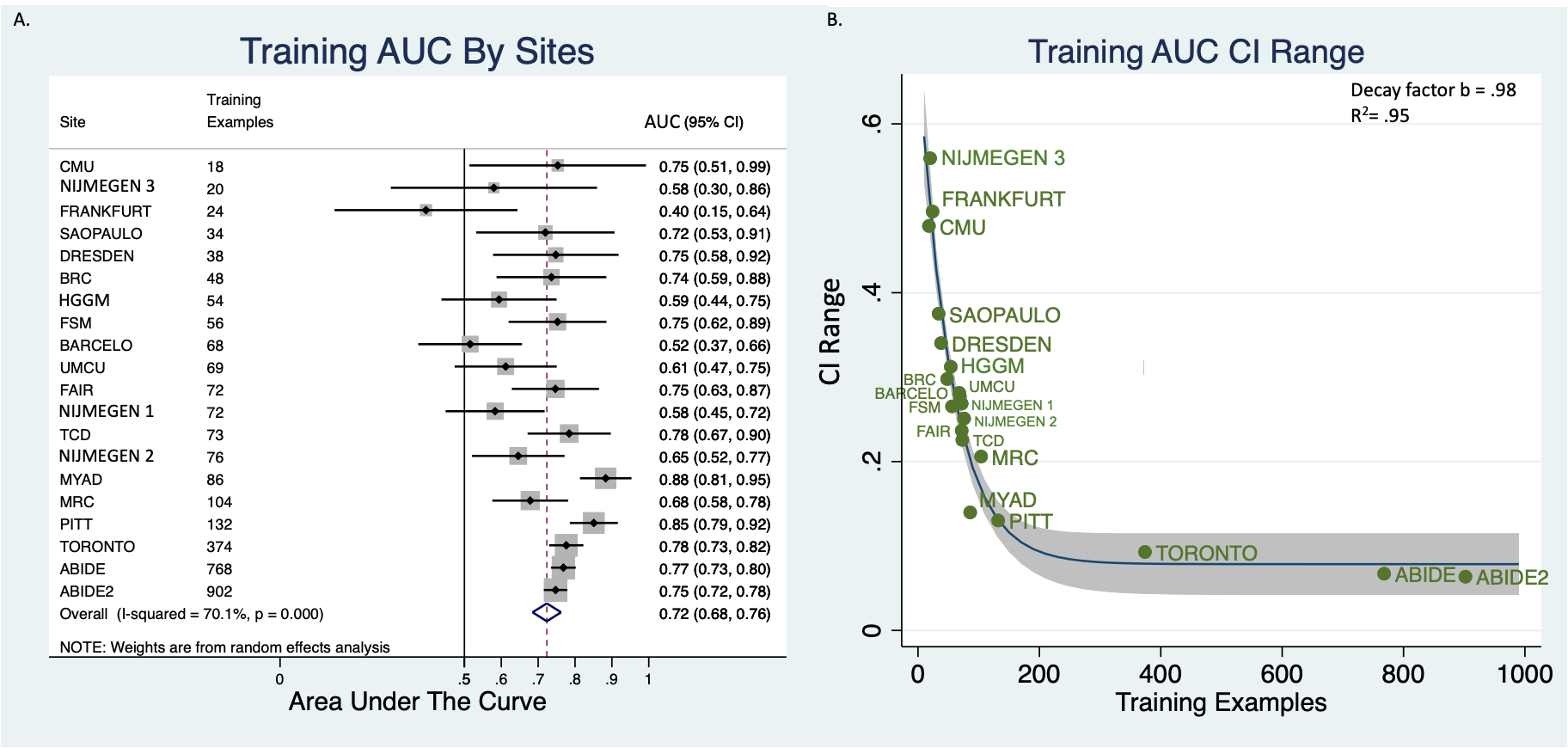


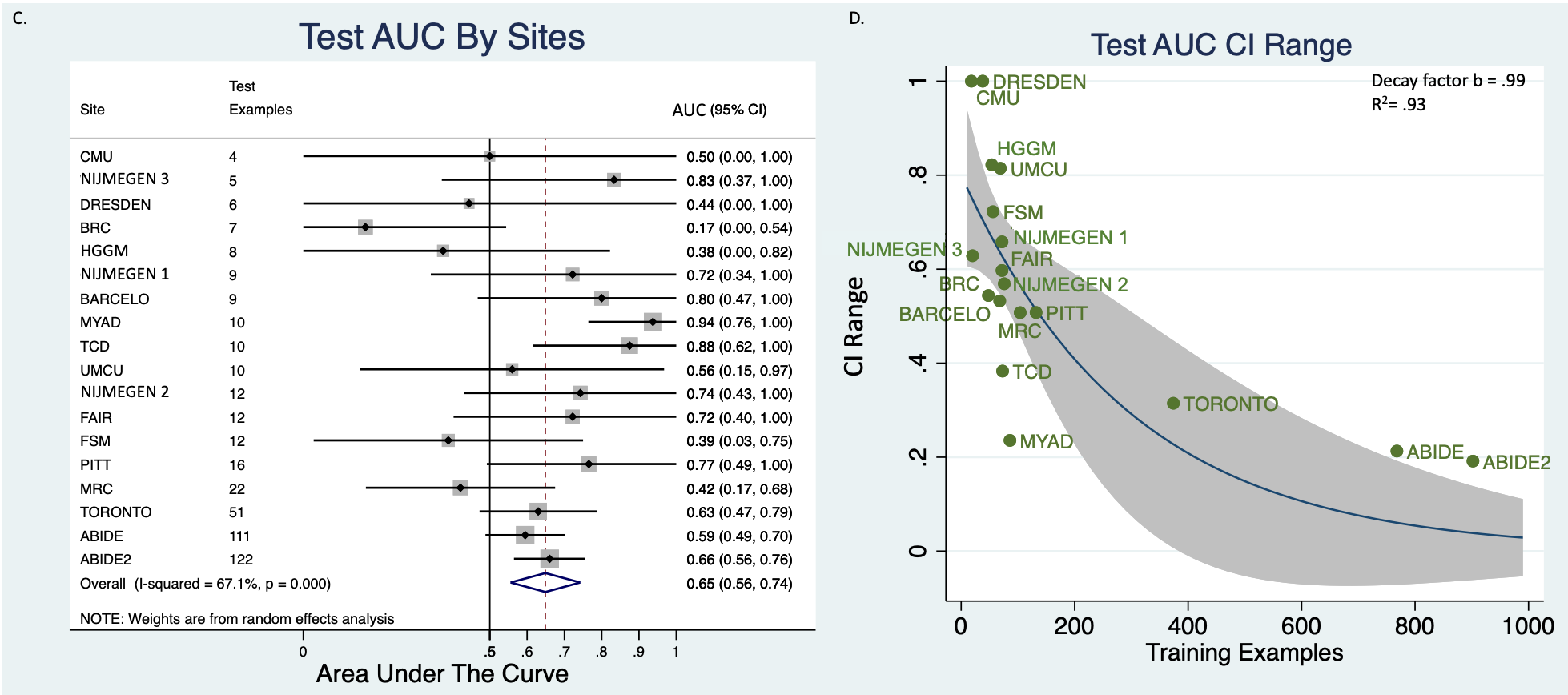


Fig S3. Training (A) and Test set (C) AUCs were shown for each site with the 95%CI. Sizes of the shaded squares are proportional to the samples sizes. Overall AUCs are shown as the red line using a random effect model weighted by sample sizes. The 95% confidence interval (CI) range was found negatively associated with training sample sizes. In B and D, the CI range for the training (B) and test (D) sets were both fitted in an exponential decay curve.

## Fig S4. Feature Importance Analysis. **A.** Feature importance correlations across different base models. **B.** Scatter plot of feature importance scores between the linear models and non-linear models.

1.

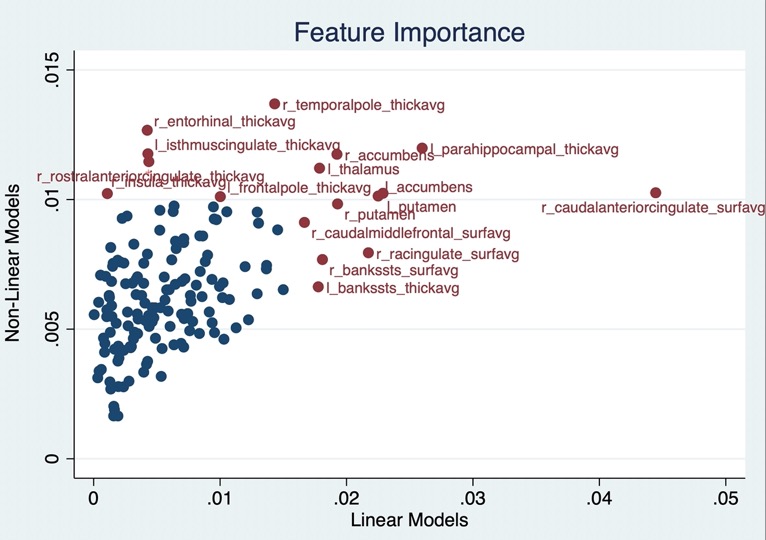


Fig S4. Feature Importance Analysis. A. Feature importance correlations across different base models. B. Scatter plot of feature importance scores between the linear models and non-linear models.
